# Obtaining informed consent in veterinary clinical trials mini review

**DOI:** 10.3389/fvets.2024.1426014

**Published:** 2024-06-25

**Authors:** Carol E. Frederick

**Affiliations:** Cornell University Hospital for Animals, Clinical Trials, Cornell University, College of Veterinary Medicine, Ithaca, NY, United States

**Keywords:** veterinary, clinical research, best practices, client consent, consent

## Abstract

In September 2023 the United States Food and Drug Administration (FDA) released draft guidance for comment about how informed client consent for companion animal clinical trials should be obtained. This guidance has the potential to substantially change how informed consent documents are written and presented to clients in the veterinary community. It provides specifics not only about how to obtain informed consent from owners but also the timeframe within which consent should be obtained, the formatting and language in the consent forms, and details on elements that are required to be in these consent forms. These changes will involve additional efforts by investigators to ensure compliance yet might lead to increased owner compliance and higher enrollment in clinical studies with subsequent benefits for all.

## 1 Introduction

The concept of clinical trials is very old. For example, there is documentation of a study from the 1750s by the British Naval surgeon James Lind on how to treat scurvy. The legislation that governs the conduct of clinical trials in humans is much newer, however. Statistically based clinical trials became a critically important part of evidence-based medicine in the United States following World War II (1).

Despite public perceptions of the importance of clinical trials, and published documentation of their utility, the FDA did not legally require clinical studies prior to routine prescription of medical therapies, devices, and pharmaceuticals until the thalidomide crisis in 1962. This drug was not FDA approved in the United States for pregnant women despite the fact it was marketed internationally as a morning sickness treatment, yet many doctors who had been provided with samples subsequently gave them to their patients without telling them the drug was experimental. Subsequently, an increasing number of regulations and guidelines have been issued by the FDA, refining definitions and requirements for the conduct of clinical trials (1).

In January 2017, the Department of Health and Human Services launched updated regulations for the protection of human research subjects. These were amended in January and June of 2018 and are now referred to as the 2018 Common Rule (2) and remains current policy guiding the informed consent process for human subjects.

Until the release of this draft guidance in September 2023, official guidance or established guidelines for obtaining informed client consent for veterinary clinical trials were unclear and lacked detail. A guidance document describing good clinical practice in veterinary clinical trials, released in 2001, defined informed consent as, “A documented process by which an owner, or owner’s agent, voluntarily confirms the owner’s willingness to allow their animal(s) to participate in a particular study, after having been informed of all aspects of the study that are relevant to the decision to participate” (3). The document contained no further guidance on informed owner consent for veterinary clinical studies and hence investigators have generally needed to look to the human subject laws and guidelines for guidance.

The veterinary community has made a number of efforts to standardize clinical trials conduct, as seen in a few papers, such as Quality assurance and best research practices for non-regulated veterinary clinical studies (4), Conduct, Oversight, and Ethical Considerations of Clinical Trials in Companion Animals with Cancer: Report of a Workship on Best Practice Recommendations (5), a review that addressed consent (6), and an additional paper discussing the importance of informed consent published in the United Kingdom (7).

With the September 2023 FDA released draft guidance to the veterinary community there is now official guidance (although not yet regulation) to follow.

### 1.1 Universal consent form requirements

The following components should always be included within an informed consent form. These are the same as those required by the 2018 Common Rule for human participants. Animal owners should be provided with a description of the benefits and details of any compensation for their participation. If the participating animal is not expected to directly benefit from study involvement then this should be clearly stated. There should also be a clear statement that owners may withdraw their consent at any time, and that the pet may be removed from the study at the discretion of the investigator with or without the owner’s consent. If there are any potential consequences for the patient that could result from early withdrawal from the study, they should be clearly outlined.

All risks associated with participating in the study should be clearly listed, including any safety data or relevant findings in pilot data or published literature. The risks listed also need to include those associated with study procedures including sedation, anesthesia, or surgery, even if those are also outlined in the general consent for treatment. If there are any risks to the owner by handling an investigational drug or product, or their pet’s waste after administration of a study intervention, they must be detailed in the consent form.

Owners should know that if there are any significant new findings that affect the validity or conduct of the study, that they will be provided with the information in a timely manner. These findings could include unexpected adverse events, an increase in adverse events compared to what was described in the consent form, a lack of effectiveness or new data from other unrelated studies that affect the trial.

A statement describing the extent of confidentiality of records identifying the owners should be included. This statement should also include how data will be used and that the confidentiality described will not be affected by any decisions the owner makes.

All owners should sign the consent form, acknowledging they have been provided with this information.

### 1.2 Readability requirements

The Office for Human Research Protection and the FDA have laws (2018 Common Rule) (2) and guidance (Informed Consent: Guidance for IRBs, Clinical Investigators, and Sponsors) (8) respectively that stipulate the language that must be used in consent documents. While the 2018 Common Rule is very clear that the information “must be organized and presented in a way that facilitates comprehension,” it does not specify a readability level target (2). There are multiple studies on human participant consent forms that report common readability statistics in consent forms cluster around the 9th-12th grade level. A recent study that assessed veterinary consent forms suggests these are typically written at the same level (9).

For the veterinary community the FDA has gone a step further and specified that all consent forms should be written at a 5th-6th grade reading level. It is widely stated the average American reads at a 7th-8th grade level, yet The National Literacy Institute states that 54% of Americans read below a 6th grade level and that 20% are functionally illiterate, reading below a 5th grade level (10).

Beyond grade level, the FDA also makes recommendations on the formatting of consent forms including using bulleted lists, using the active voice, writing short sentences with simple structure, use of wide margins, and using legible fonts and font size. They further counsel that justified text, medical jargon, italics, and use of all capital letters should be avoided. The FDA guidance recommends using statements that clients have been provided with all the relevant information rather than statements that indicate that clients have understood all relevant information.

### 1.3 Coercive language

The draft guidance states that all potentially coercive or persuasive language should be removed from client consent forms. Persuasive language is a potentially biased form of writing that often emphasizes only one perspective. In the context of clinical trials, such language might emphasize the possible benefits of the trial without adequately describing the potential risks involved with participation. Persuasive language often involves appealing to the reader’s ethics, morals, emotions, or logic to encourage or convince.

### 1.4 Eligibility and enrollment guidelines

The guidelines also suggest that “enrollment of animals owned by investigators, employees or relatives of investigators or the sponsor, or any person with any direct or indirect interest in the outcome of the study” should be avoided. This is sensible for workplaces with smaller numbers of employees, or those where the investigator also has influence over salary, promotion or employment conditions, and to remove the possibility of the data being affected by those who have a direct interest in the outcome or a monetary interest in the success of the drug. In large institutions, such as veterinary teaching hospitals or large practices, however, this could be unnecessarily detrimental by depriving studies of willing participants where there is little or no likelihood of duress or undue influence by study investigators. For instance, incorporating such language might preclude participation by employees in entirely separate departments or by students in the case of veterinary schools. No employee should be required to participate in any study, but use of language that maximizes the pool of potentially willing participants is reasonable provided there are no adverse consequences for non-participation.

### 1.5 Description of the clinical study

An informed consent form should contain a description of the study objectives. In addition, the new FDA guidance document also recommends that treatment groups are fully described, including how many animals are to be enrolled and provides an estimate of the odds of being in each treatment group. Further, rather than highlighting only what the investigational treatment is, the guidance document suggests also describing what represents standard of care treatment to better highlight the difference between usual care and that provided to animals enrolled in the study. Owners should be clearly informed of their responsibilities while their pet is participating in the study. These responsibilities might include masking or blinding, the timeline of all rechecks, requirements to complete forms or questionnaires, and what, if any, restrictions will be placed on them or their animal.

### 1.6 Timing of the consent process

The legislation around timing of consenting human patients to a study states that consent should be obtained “only under circumstances that provide the prospective subject or the legally authorized representative sufficient opportunity to discuss and consider whether or not to participate and that minimize the possibility of coercion or undue influence” (2). There is no specific amount of time specified in this law. For companion animals the FDA now interprets that statement to mean that, when possible, owners should be provided consent forms at least 12–24 hours prior to being asked to make a decision. Clearly, this will not be possible for studies that seek to enroll patients presented on an emergency basis where the study might seek to collect patient data or samples prior to the initiation of therapy. In such instances, particular care must be taken by the study investigators to provide clients with the maximum amount of time available and to be scrupulous in avoiding pressuring clients into participation.

### 1.7 Actionable recommendations

Currently, the draft FDA guidance document is just that – draft guidance and does not represent legislation. It is reasonable to presume that few changes to the document will result from public consultation on the draft of the official guidance but confirming that is true when the final document is published is advisable. With that in mind, it makes sense that study investigators consider the document as representing best practice and are recommended to adhere to as many of the guidelines as possible. In some cases, this may require substantial revisions to existing study documents and client consent forms.

It is recognized that consent forms will necessarily continue to vary widely with study design. There is a large difference in the potential for risk and reward for an interventional study compared to one that involves collection of a small additional blood sample on a single occasion. Irrespective, it may be prudent to design a modifiable consent form template that contains all the required elements to prevent omissions while also saving investigators time. Several readily available resources exist to check the readability of your documents including tools built into commonly used word processing software that can check spelling and grammar and provide the Flesch Reading Ease score (11) and the Flesch–Kincaid grade level (12).

### 1.8 Summary

While the new FDA guidance may require significant rewrites of current consent forms, this ultimately represents an opportunity for those engaged in clinical research to improve study participation. Clinical trials in all species have long suffered from difficulty recruiting sufficient study subjects in a timely manner. Rewriting consent documents to be more accessible to the average reader and providing more time for clients to consider the documents may significantly increase study participation.

## Author contributions

CF: Writing – original draft, Writing – review & editing.
